# A primacy code for odor identity

**DOI:** 10.1038/s41467-017-01432-4

**Published:** 2017-11-14

**Authors:** Christopher D. Wilson, Gabriela O. Serrano, Alexei A. Koulakov, Dmitry Rinberg

**Affiliations:** 1 NYU Neuroscience Institute, New York University Langone Medical Center, New York, NY 10016 USA; 2 Cold Spring Harbor Laboratory, Cold Spring Harbor, New York, NY 11724 USA; 3 Center for Neural Science, New York University, New York, NY 10003 USA

## Abstract

Humans can identify visual objects independently of view angle and lighting, words independently of volume and pitch, and smells independently of concentration. The computational principles underlying invariant object recognition remain mostly unknown. Here we propose that, in olfaction, a small and relatively stable set comprised of the earliest activated receptors forms a code for concentration-invariant odor identity. One prediction of this “primacy coding” scheme is that decisions based on odor identity can be made solely using early odor-evoked neural activity. Using an optogenetic masking paradigm, we define the sensory integration time necessary for odor identification and demonstrate that animals can use information occurring <100 ms after inhalation onset to identify odors. Using multi-electrode array recordings of odor responses in the olfactory bulb, we find that concentration-invariant units respond earliest and at latencies that are within this behaviorally-defined time window. We propose a computational model demonstrating how such a code can be read by neural circuits of the olfactory system.

## Abstract (web summary)

Odor identity remains stable despite changes in concentration yet the neural mechanisms are relatively unknown. Here the authors test a primacy coding model using an optogenetic masking paradigm in mice to show that a set of earliest activated receptors are sufficient to make decisions about odor identity across concentrations.

## Introduction

**Fig. 1:**
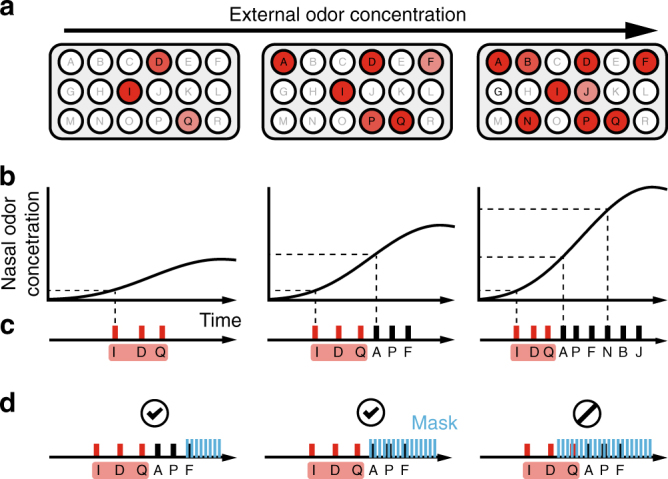
Primacy coding of odor identity. **a** Schematic presentation of the patterns of glomerulus (OSN) activation for three concentrations of the same odor. The total number of active glomeruli increases with an increase of odor concentration. **b** The temporal profiles of the odor concentration in the nose during inhalation for three concentrations of the presented stimuli. Dashed lines represent concentration thresholds for representative glomeruli (horizontal) and corresponding response latencies (vertical). **c** Temporal sequence of glomeruli activation for three different odor concentrations. **d** Optogenetic mask schematic demonstrating the effect of the optogenetic mask on temporal sequences when presented late and early relative to odor-evoked temporal pattern

A substantial computational challenge for the olfactory system lies in resolving odorant identities despite fluctuations in odor concentration arising from proximity to odorant source, air turbulence, and natural breathing^1–3^. Odorants are sensed by olfactory sensory neurons (OSNs), each expressing one out of a large family of olfactory receptor (OR) genes^4^. Axon terminals from OSNs expressing the same OR gene converge in a few discrete structures in the olfactory bulb (OB) called glomeruli. Odorants evoke responses in an ensemble of glomeruli to create a combinatorial representation of odor identity^5, 6^. This representation varies not only across odorants, but also across concentrations of a single odorant (Fig. 1a)^7–9^. Low concentrations of odorant evoke activity in only the most sensitive glomeruli, while increases in concentration result in recruitment additional less sensitive glomeruli. Despite this variability, odors’ qualitative identities are preserved across a range of concentrations^2, 10, 11^.

In several sensory systems, including olfaction, neurons have been shown to convert the strength of excitatory input into latency of response^12^, and it has been hypothesized that ORs with high affinity will depolarize OSNs earlier than those with low affinity^13–15^. This emerges as a result of multiple processes, including intracellular signal integration^16^ and temporal dynamics of odorant concentration^17–19^. For air breathing animals, sniffing determines the temporal dynamics of odorant concentration in the nose, resulting in an affinity-defined sequence of OSN recruitment. While these sequences of recruitment vary across odorants, they have been shown to be mostly concentration invariant, as changes in concentration preserve temporal rankings of ORs with different affinities and these latencies are proposed to encode information about odor identity^15, 20, 21^.

Here we propose a strategy for concentration-invariant odor identification which uses a few earliest activated glomeruli in a coding scheme we call “Primacy Coding”. We assume that the first few glomeruli activated during a sniff are those receiving input from the most sensitive ORs for a given odorant. We propose that solely the members of this small set of early glomeruli encode odor identity. While this primary set varies between odorants, we assume that it is mostly preserved across concentrations of the same odorant. As concentration is increased, responses of less sensitive glomeruli are recruited later than the primary set, thereby maintaining the members of the early set and preserving encoded odor identity information (Fig. 1b, c).

One of the central predictions of primacy coding is that animals use early “slices” of odor-evoked neural activity to define odor identity independently of the remainder of the pattern of evoked activity. To test this hypothesis, we developed an optogenetic masking paradigm in which we could create a temporally controlled masking stimulus during an odor discrimination task (Fig. 1d). Through delayed triggering of this optogenetic masking stimulus relative to inhalation onset, we could preserve early epochs of odor-evoked information while making the overall combinatorial code unreliable through the activation of a large, heterogeneous subset of OSNs. We reasoned that if odor identity can be defined using only a small subset of early-responding primary glomeruli, our mask should not impair odor identification as long as it is initiated after this identity-defining subset. Conversely, activation of the mask before this initial subset of glomeruli is active should impair odor discrimination.

## Results

### Optogenetic masking of late odor-evoked activity

**Fig. 2:**
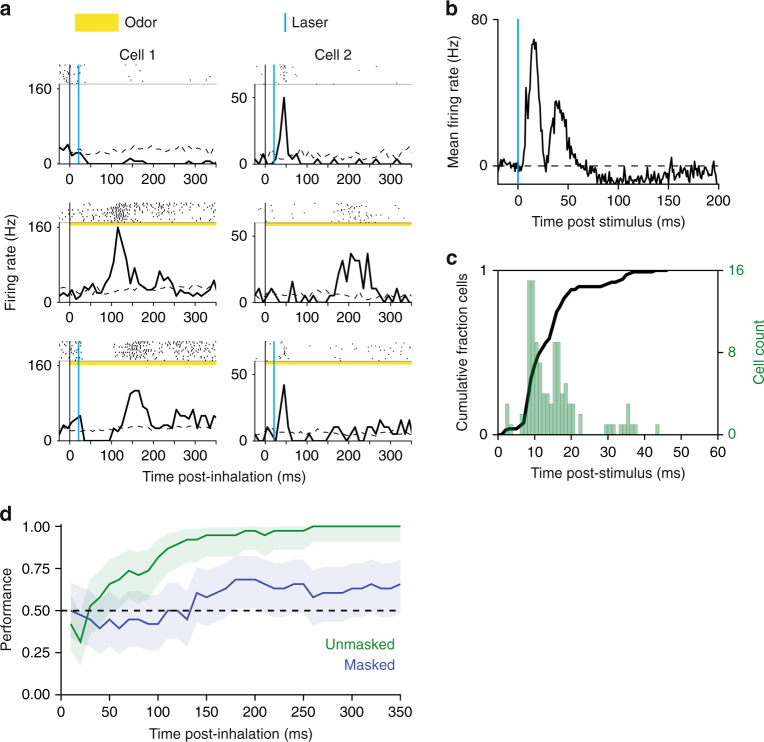
Neural response to optogenetic mask and effect on odor information. **a** Example raster and PSTH for two example MT cells. Inhalation onset corresponds to *t* = 0. Top: Example MT cell responses to optogenetic mask (25 mW, ON:2 ms—OFF:8 ms—ON:2 ms), light stimulation at 20 ms post-inhalation onset. Middle: Odor response (left: 2-hydroxyacetophenone, right: alpha-pinene). Bottom: Odor plus laser mask. **b** Baseline-subtracted mean of PSTHs for laser-responsive units (*n* = 113). **c** Cumulative distribution function (black line) and histogram of units’ response latencies to masking light stimulus. **d** Linear classifier cross-validation results for unmasked data for two odor presentations (limonene, pinene). Responses to unmasked odor presentations were time-binned (10 ms bins) and used to train support vector machine (SVM) classifiers. For each time point, SVMs were trained using the response vector inclusive of bins from *t* = 0 to that time. Each classifier’s performance is described by cross-validation on unmasked trials (blue) and testing on masked trials (green). Shaded areas indicate 95% confidence interval (Clopper–Pearson method)

To produce the mask stimulus, we delivered 2 light pulses (25 mW, 2 ms duration, 10 ms inter-pulse interval) to the olfactory epithelium in both nostrils of the transgenic mouse expressing channel rhodopsin-2 (ChR2) in all OSNs^22^. The light was delivered via optical fiber stubs implanted above the olfactory epithelium. To characterize the neural response to the mask stimulus, we recorded light and odor-evoked activity of mitral-tufted (MT) cells (*n* = 119: 39 single-unit, 80 multi-unit) in the OB, which are the first recipient of input from OSNs (Fig. 2a, Supplementary Fig. 2A–D). The masking stimulus generated responses occurring after a short delay following light onset (mean = 11.5 ms, mode = 8 ms) (Fig. 2c). The overall mask excitatory response lasts approximately 50 ms, followed by prolonged inhibitory response until approximately 200 ms (Fig. 2b, Supplementary Fig. 2E).

How effective is the mask in eliminating odorant responses? Out of 119 recorded units, 29 responded to one of two odorants (pinene, limonene). The mask, presented at 20 ms latency post inhalation onset, modified most of the odor responses, even though odor responses typically occur later than the mask stimulus (Fig. 2a). To characterize the effect of mask on information available for discrimination, a support vector machine (SVM) was trained to classify MT cell responses to two odorants (limonene vs. pinene) with and without masking stimulus.

Only on the basis of the odor responses of 29 units, classification performance on an individual trial raised from chance level to 100% in the first 260 ms from the inhalation onset. On the masked trials, performance of the classifier stayed at chance level until ~ 180 ms post inhalation onset, and never exceeded 68% (Fig. 2d). We may assume that the mask is efficient in eliminating odor information for at least 100 ms following the mask.

**Fig. 3:**
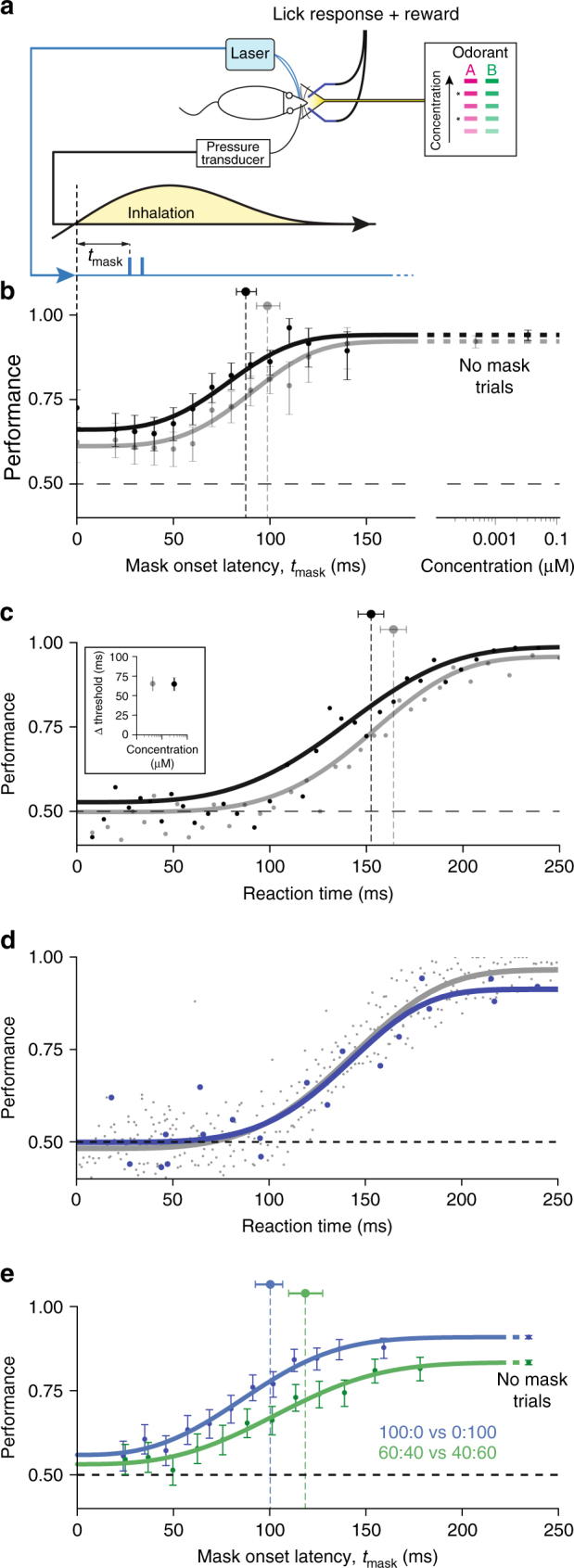
Optogenetic masking behavioral paradigm. **a** Behavioral task schematic: Mice were trained to respond to 2 odors "A" and "B" at 5 different concentrations to lick left or right water spout. Mask timed to the onset of the first inhalation after odor delivery was presented during subset of trials for two concentrations (asterisks). **b** Discrimination performance vs. mask latency for odors (2-hydroxyacetophenone and eugenol) at two concentrations. Mask stimulus presentation was initiated on the first inhalation of odorant and after the mask onset latency, *t*_*mask*_, had elapsed. High concentration annotated in black markers, low concentration in grey. Error bars indicate 95% confidence interval estimates. Weibull fit to the data indicated with thick lines. Markers above performance curves indicate Weibull threshold latency values for each fit with 95% confidence interval estimates. **c** Mouse reaction time vs. performance in unmasked stimuli of two concentrations (as above). Dots indicate the data binned into bins of 125 trials. Lines indicate Weibull fit. Inset: difference between mask and reaction time threshold latencies. **d** Mouse reaction time vs. performance for unmasked trials (gray) compared with late masked trials with *t*_mask_ >  = 100 ms (blue) for the same data set. Points represent bins of 50 trials each. **e** Performance vs. mask latency for discrimination of pure (blue) carvone enantiomers vs. mixtures made with those odorants (green)

To test the effect of the mask on behavior, mice (*n* = 3) were trained in a head-fixed 2-alternative choice paradigm to discriminate between two odorants (eugenol and 2-hydroxyacetophenone) for a water reward (Fig. 3a). To ensure that decisions were based on odor identity and not intensity, we scrambled the odorant concentrations by presenting five concentrations within a two order of magnitude range. On probe trials within the session, the optogenetic mask was presented with target odor. The probe trial structure was used to prevent animals from adopting a novel strategy to overcome the effect of the mask. The animals’ performance was strongly affected by the masking stimulus when it was initiated between 0 and 50 ms after inhalation onset (Fig. 3b and Supplementary Fig. 1E). The presence of the mask lowered the mean performance at these early latencies to almost chance level of 56% compared with the unmasked performance of 92% (at odorant concentration 1 μM). As the onset latency of the mask is increased to latencies greater than 50 ms, performance in the odor identification task recovers and approximates the unmasked asymptotic performance at ~100 ms.

### Scaling of integration time with odorant concentration

Changes in odorant concentration should change the absolute timing of OSN recruitment and, thus, affect the timing of odor percept formation in our task. We tested this prediction by fitting a Weibull generalized linear model (see “Methods” section) to masking data for two concentrations of odorant and comparing the thresholds of these fits. A 10-fold decrease in concentration delays the recovery of performance in masking trials by 13.3 ms (100.3 vs. 87.0 ms, bootstrapped 95% confidence intervals [94.2, 105.9] and (81.8, 91.5), *p* = 0.048 (one-tailed Monte Carlo test, *R* = 50,000). As expected, odor percepts are formed later for lower odorant concentrations, likely due to delayed recruitment of OSN activity^12^. Very similar dependencies have been observed for other odor pairs and 5 other mice (Supplementary Fig. 1D).

Reaction time (RT) has been used extensively to determine the timing of sensory processing and decision-making based on sensory stimuli. The dependence of performance on both mask latency and RT are qualitatively similar, except that the reaction time curve is shifted by ~66 ms. This relationship is similar for both concentrations tested here (65.5 and 66.2 ms, Fig. 3c, Insert). The concentration-dependent shift in RT vs. performance can be wholly explained by the shift observed in our masking paradigm, indicating that peripheral encoding of odor information limits the timing of olfactory decision making. The observed delay between the RT and mask can be ascribed to a motor delay that is constant across concentrations.

Mask trials may promote the use of a novel strategy to utilize early information. To address this issue, we performed two analyses. First, we compared reaction time in unmasked trials and trials where the mask was presented late (t ≥ 100 ms) and had minimal effect on performance (Fig. 3d and Supplementary Fig. 1G). The Weibull fits to the dependencies of performance on reaction time in both cases are nearly identical. Second, we see no effect of learning on mask trials between early and late behavioral sessions, comparing the performance on the first and last 20 masked trials across all sessions (Fisher-Exact test *p* > 0.05). (Supplementary Fig. 1F). Together, these results provide evidence that randomly presented masking trials do not change the animal decision strategy and that animals typically use early evoked information in the odor discrimination tasks.

### Scaling of integration time with odor contrast

Does primacy coding strategy apply only to simple tasks? To make the behavioral task more difficult, we trained animals to discriminate between mixtures of carvone enantiomers (60:40 vs. 40:60). Using mixtures to reduce the contrast between stimuli decreases discrimination performance relative to pure carvones (83.3 vs. 90.8%) and delays the mask threshold time (118 ms vs. 101 ms) (Fig. 3e). As with the latency shift between concentrations of odorant, the shift due to decreased contrast is consistent with the measure of reaction time vs. performance (Supplementary Fig. 1H). To generate an equivalent level of performance in the more difficult mixture discrimination task, sensory information must be integrated by the subject over a longer period of time. However, the extension in integration time is relatively small on the scale of the length of a sniff, demonstrating that animals are still using early information to make this more difficult discrimination.

### Neural correlates of concentration-invariant primacy code

**Fig. 4:**
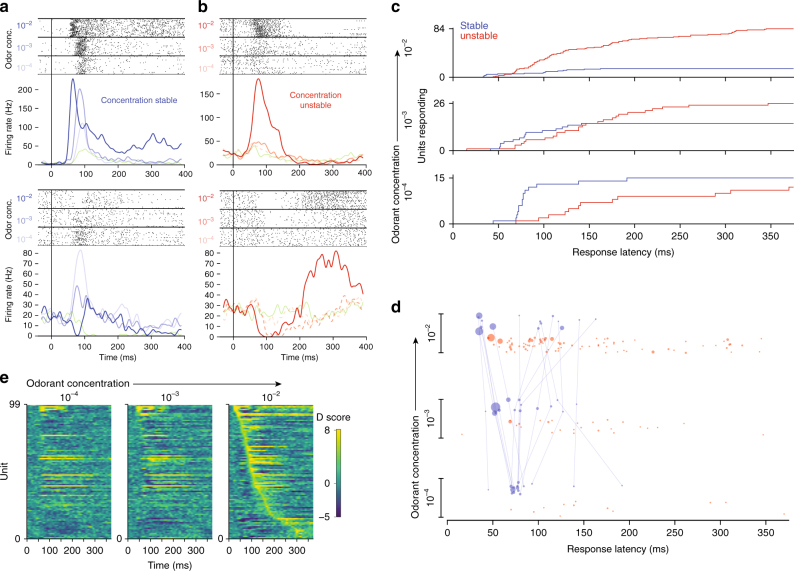
Concentration invariant MT cell response latencies correspond with behavior. **a** Example raster and PSTH of two MT cells, which were responsive to odorant across 3 orders of magnitude concentration. *t* = 0 corresponds to inhalation onset. **b** Same plots for MT cells which respond to only a subset of concentrations. Dashed PSTH lines represent responses that did not cross the threshold for significance (see methods). **c** Cumulative distributions of MT cell response latencies. **d** Response latencies of MT cell-odor pairs across concentrations for concentration-stable and unstable units. Units’ responses across concentrations are connected. Area of each point in the plot is proportional to the Cohen’s D-score of the response over non-odor response (see “methods” section). Point positions are randomly jittered on the concentration axis for visibility. **e** Odor responses sorted by units’ response latencies at the highest concentration of odorant. Responses are represented as D-scores, where positive and negative scores are excitation and inhibition relative to blank inhalation responses

Our behavioral results define the temporal window in which odor information is integrated. To determine if primacy codes exist in the OB at timescales consistent with our behavior, we recorded responses of 338 MT cells to 3 odorant concentrations spanning a range of 2 order of magnitude. The primacy coding model predicts the existence of MT neurons that display excitatory responses to odor across a wide range of concentrations in a narrow temporal window at the beginning of the sniff. Among 119 units which exhibit excitatory responses to odorant α-pinene, 15 responded to all three concentrations, a subset that we called “concentration-stable” (Fig. 4a). The remaining 104 units responded only to subset of concentrations, which we called “concentration-unstable” (Fig. 4b). As expected, a number of responsive units grew as concentration increased (Fig. 4c). While the population response at low concentration was dominated by concentration-stable units, unstable units were 3.7 times more numerous at the highest concentration tested.

According to our primacy coding model, the identity of early, stable units that are responsive across concentrations represent odor identity. The stable population’s mean response latency was shorter than the unstable population at all concentrations (conc 10^−3^: 86.8 ms vs. 177.2 ms, *p* = 0.0003; conc 10^−2^: 82.7 ms vs. 148.2 ms, *p* = 0.0071; and conc 10^−1^: 85.7 ms vs. 149.0 ms, *p* = 0.0249; one-sided KS test). While the latencies of the unstable and stable populations overlapped, a subset of stable units responded earlier than all unstable units at the highest concentration, which is consistent with the primacy coding model (Fig. 4d). The latencies of these stable units scaled with concentration, as predicted by the model and behavioral result. The mean latency shift between concentrations for this early, concentration-stable subset was 15.5 ms, (*σ*: 5.5 ms). This is comparable to the timing shifts in behavioral identification across concentration (13.3 ms). The stable population’s activity encoded odor-identity information and was not representative of non-specific odor responses; only one of these concentration-stable units was responsive to another odorant tested, α-limonene.

Importantly, we find that latencies and even latency relationships are not preserved across concentrations in awake animals, although this has been observed in anesthetized animals (Fig. 4d)^20^. Instead, both absolute and relative latencies of stable units' responses shift dramatically as concentration increases, clustering into early and late subsets at the highest concentrations. We observed that the excitatory responses of the late set of stable cells was preceded by transient inhibition at high concentrations (Fig. 4a lower panel, 4e). This transient odor-evoked inhibition was observed widely in our recorded population (Supplementary Fig. 3F, H), and we hypothesize that it is due to lateral and feedback inhibition in the OB. For late stable units, we predict that earlier members of the MT population drive inhibition arriving earlier than or in coincidence with feedforward excitation. This shunts the neuron’s initial excitatory response, causing an apparent increase in response latency. As a result, we conclude that these units are not in the primary set, as it is obvious that earlier members of the population must precede them to generate the network activity responsible for this inhibition. So, while short latency is predictive of concentration-invariance, the relationships of response latencies within our recorded population as a whole are not conserved across concentrations and are unlikely to encode odor identity.

### Read out of primacy code by a model neural network

**Fig. 5:**
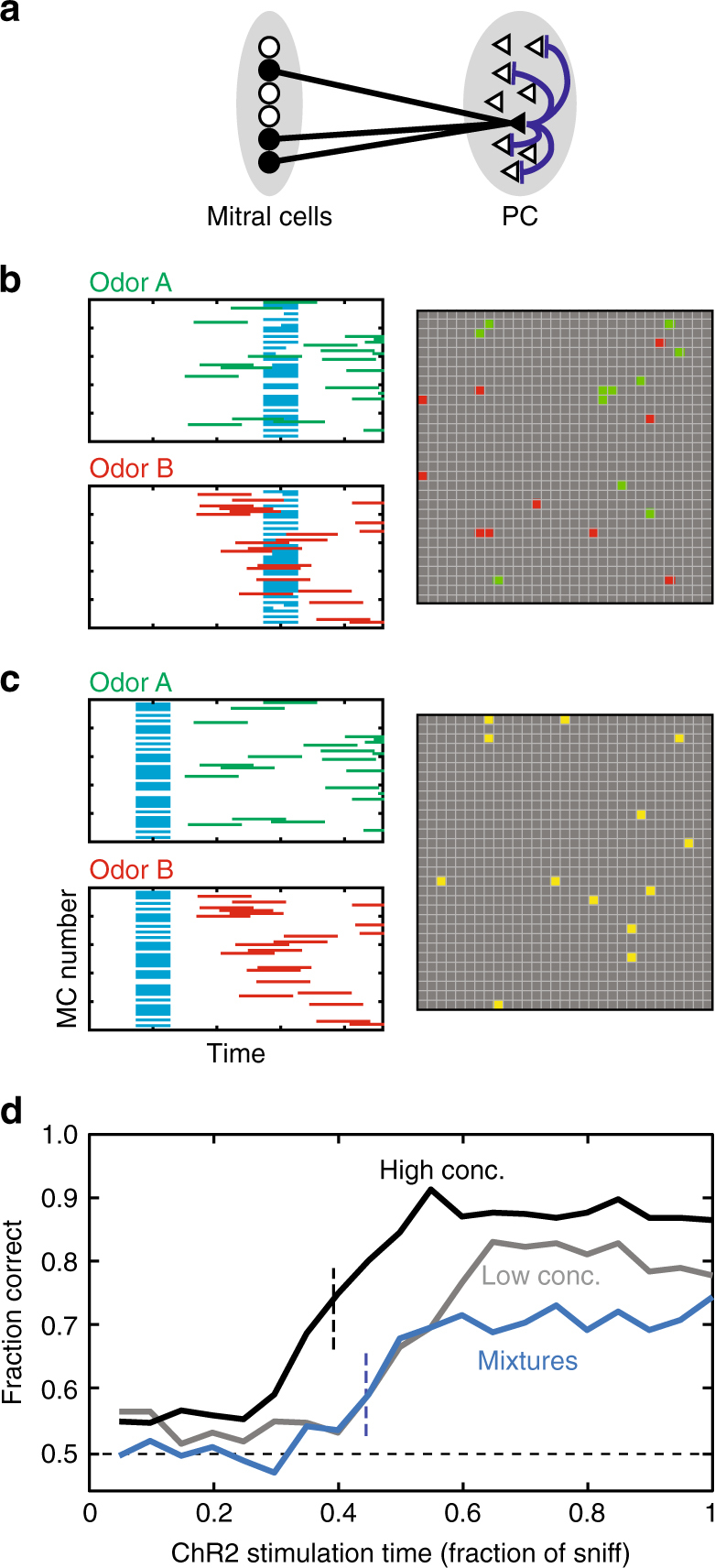
Computational model of primacy decoder can explain behavioral effects of mask. **a** The schematic of the network included in the computational model. Pyramidal neurons in PC receive random excitatory connections from MT cells (black lines) and connected to a random subset of other pyramidal neurons via inhibitory connections (blue lines). The inhibitory connections are responsible for blocking of the inputs to PC after the initial (primary) representation is formed. **b**, **c** Responses of MCs (100 out of 300) and PC cells for late (**b**) and early (**c**) masks. MCs respond to Odor A, Odor B, and ChR2 (green, red, cyan). PC responses at the end of sniff cycle (gray squares) are shown for two odors in the same panel. For the early mask (**c**), PC responses are indistinguishable for two smells (yellow). **d** Discrimination success rate as a function of mask latency for three conditions: odors A vs. B—high concentrations (black line), odors A vs. B–low concentrations (gray line), odor mixtures 60% A + 40% B vs. 40% A + 60% B (blue line). Low/high concentration conditions were introduced by expanding/compressing the timing of MC odorant-related inputs within the sniff cycle. Mixtures were introduced by combining together high conc. sequences of inputs corresponding to odors A and B. The temporal dependencies reproduce the basic qualitative features of data (Fig. 2b, e), including concentration-dependence of the error rate at different ChR2 timings

What elements of olfactory networks are sufficient to process primacy information? How does mask affect odor recognition? Our behavioral data show an incomplete suppression of animal performance at small masking delays (Fig. 3b). In a more difficult discrimination task, the effect of mask is slightly delayed compared to easier tasks (Fig. 3e). Are these features expected within the primacy coding mechanism? To address these questions, we developed a computational primacy decoding model based on known features of the OB and piriform cortical (PC) circuits. First, our model includes random feedforward connectivity between the OB and PC which provides the basis for coincidence detection of MT cell activity arriving early in the sniff cycle. Second, our model includes random recurrent inhibitory circuits in the PC (blue in Fig. 5a). The role of this connectivity is to suppress late arriving, “non-primary” input to PC neurons. This architecture is consistent with observed global and broadly tuned inhibition in the PC^23^ and has been used as a feature of network models for the processing of fine temporal information in the PC, including the proposed mechanism for coincidence detection^24^. Finally, we assumed that PC neurons have a memory property such that once activated, they can maintain the persistent activity in order to retain the odorant representation in the network until the initiation of action. Although our computational model does not explicitly specify the mechanism of persistency, it has been hypothesized to emerge from voltage dependent synaptic channels, such as NMDAR or GABA_B_ activating KIR channels^24^. Overall, our network included randomly connected 300 MC and 1000 PC neurons.

Our model provides insights into the mechanism of optogenetic suppression of the animal’s performance for early delivered masks. We simulated odor-evoked activity in MT cells as a random spatiotemporal pattern and the mask as synchronous activity independent of odor (Fig. 5b, c). When the mask follows initial odor-evoked activity, it does not affect odorant-dependent activity patterns in PC (Fig. 5b), due to the broad inhibitory network, which suppresses excitatory inputs from the late mask or odorant-dependent inputs. In contrast, when the mask precedes odor activation, a pattern of activity emerging in the PC is not odorant-dependent and is different from those of the odorants alone (Fig. 5c). This light-evoked pattern can be viewed as a new percept that is unrelated to the original odors.

Our model qualitatively explains an incomplete suppression of discrimination for early masks (*t* < 0.2) (Figs. 3b and 5d, black and gray lines). This occurs due to the presence of noise: on certain trials, odor-dependent OB responses are more robust than on average, causing failure of the mask on these trials (Supplementary Fig. 4). In case of complex mixture, the behavioral response is delayed compared to an easy stimulus (Fig. 5d, blue vs. black lines), which is consistent with experimental results (Fig. 3e). This occurs because the differences in pools of primary glomeruli emerge slightly later in the sniff cycle, as both of the stimuli have similar sets of initially activated glomeruli. This result implies that primacy read out mechanism does not take the sequence of glomerular activation into account, since such differences are expected to occur early. In a version of the model sensitive to the activation sequence, the delay in performance for complex tasks is strongly reduced (Supplementary Fig. 5). Overall, our computational model confirms that the experimental data is consistent with the identity of primary MT cells rather their recruitment order being important for the coding of odor identity.

## Discussion

We demonstrate here that the earliest evoked neural activity can be used to make olfactory decisions and demonstrate that neural activity in this time window may encode odor identity across concentrations through primacy coding. Previous behavioral studies demonstrate that early epochs of odor-evoked activity are sufficient for the detection of odorants^25, 26^ and concentration discrimination^27^ in rodents. However, while these studies allude to fast coding of olfactory stimuli, it is impossible to rule out subjects’ use of intensity cues that are external to odor identity. By scrambling concentrations of odorant, we make intensity cues unreliable and encourage the use of odor identity in these experiments. Studies using activity-dependent imaging have proposed that the most sensitive glomeruli may encode individual odors^15, 28^; however, to our knowledge, our work provides the first behavioral evidence and computational support for this hypothesis.

Primacy coding and latency coding both attempt to solve the “analog match” problem odor identification through the use of timing, although they each make unique predictions. Central to both models is the expression of input strength to individual sensory neurons into spike latency relative to theta rhythm^14^ or to inhalation. Apart from the synthesis of these temporal patterns, primacy coding and latency coding differ significantly in the mechanisms by which these spatiotemporal patterns are read out. In general, latency coding describes a family of models that match a vector of response latencies evoked by an unknown stimulus with a prototype latency vector. This comparison has been modeled through a combination of a “delay line” architecture with coincidence detection^29, 30^. In these models, a decoder cell receives inputs from several glomeruli, each of which is delayed such that their signals arrive coincidentally at the decoder neuron only when a specific odor is encountered. These models imply that relative latencies of different glomeruli must scale equally with concentration to preserve coincidence, while in our data we find that both absolute and relative latencies within the population of odor-responsive MT units are not maintained across concentrations in awake animals (Fig. 4e).

As predicted by Hopfield, computational models of the OB based on the experimental data demonstrate that similarity of these latency vectors across concentrations of the same odorant are more similar than latency vectors generated by different odorants^31^. If latency vectors for different odors are evaluated in snap-shots that evolve after inhalation, divergence is predicted at short timescales ( < 100 ms) for dissimilar odorants. However, the mechanisms by which these vectors are evaluated on these short timescales has not been fully explored^24^.

Primacy coding extends latency encoding models by making the assumption that solely the earliest members of the set are representative of odors across concentrations. It proposes that temporal relationships are important only insofar as they help in identifying these early members, and predicts a basic network architecture that can decode this early information to create stable patterns in piriform cortex^44^. These patterns, by definition, are formed fast and through the use of early divergence of the spatiotemporal pattern of glomerular activity. The primacy model emphasizes the role of the set membership of early-responding neurons rather than the sequence of glomerular activation. Our experiments with mixtures in combination with computational modeling provide indirect evidence towards this prediction. Further experiments with precise temporal control of early activated glomeruli may confirm this prediction.

The primacy model sets limits for information capacity of the olfactory code. The upper bound estimate for the number of odorant identities represented by the primacy code is ~${N^p}/p!$_,_ where *N* is the number of different OR types and *p* is size of the subset used to define identity. With *p* = 5–6 and the *N* = 350 OR genes found in the human genome, the coding scheme can represent ~ 10^10^–10^12^ different odors.

The primacy model emphasizes the role of individual glomeruli for odor coding. In mice, a deletion of a single receptor TAAR4 is sufficient to abolish aversive behavior to a specific odor^32^. Studies of human genetic variability lends evidence that only highly sensitive receptors predominate in defining odor quality. Subjects with different alleles of a single OR report differences in perceptual qualities for strong ligands of the OR, while they are likely to report similar qualities for weaker ligands^33^.

The primacy model also makes specific predictions about mixtures of odorants because the earliest activity should dominate perceptual qualities of the mixture. Perceptual masking of slowly perceived odors by fast odors (temporal suppression) has been observed in human psychophysics, but warrants more attention^34^. If both odorants evoke early activity within the primary set, we predict that this combination will cooperate to synthesize a new odor percept. Finally, primacy coding does not make explicit provision for parallel processing of components in mixtures, a task where humans demonstrate poor performance^35^.

Primacy coding suggests a relatively simple solution to the complex computational problem of robust concentration-invariant representation of odorant identity in olfaction. It provides inherently rapid odor identification, a vast coding capacity and can be implemented by the architecture of the olfactory system.

## Methods

### Mice

The behavioral concentration series data were collected in 4 *OMP-ChR2-YFP* heterozygous mice (2 female, 2 male). The mixture data were collected in a separate cohort of 5 *OMP-ChR2-YFP* heterozygous mice (2 female, 3 male). Electrophysiological data to characterize masking were collected from a separate cohort of mice (*n* = 2). Five male C57B/6 mice (Jackson Labs) were used for concentration series electrophysiology. Subjects were 8–12 weeks old at implantation and were maintained on 12hr light–dark cycle in isolated cages after implantation. All procedures were approved by the IACUC of NYULMC in compliance with the NIH guidelines for the care and use of laboratory animals.

### Sniff recording

Sniff was monitored via intra-nasal pressure. An 8 mm long, 21-gauge cannula was implanted into the anterior dorsal recess. Total insertion depth from the surface of the nasal bone was 1.5 mm. Pressure change relative to atmospheric pressure was measured using a pressure sensor (24PCEFJ6G, Honeywell) and amplified (AD620, Analog Devices). A Schmitt (dual-threshold) trigger was used to define inhalation and exhalation onsets in real time on an Arduino microcontroller. For concentration series electrophysiology, respiration was measured using an externally mounted pressure sensor placed in front of the subjects’ nares.

### Surgery

Mice were anesthetized using isofluorane gas during surgery. The head bar, pressure cannula and optical fibers were implanted in a single surgery. The nasal cannula was implanted in a small hole in the anterior nasal bone and affixed with glue and dental cement. The optical fibers were implanted bilaterally in two holes drilled in the posterior nasal bones and affixed using the same technique. Two small screws (size #000–120 × 0.0625”, Small Parts, Inc.) used to stabilize the implant and provide electrical connection for lick detection were implanted in the skull at a location approximately corresponding with S1 cortex. Animals were allowed to recover for at least 3 days before water deprivation.

### Stimulus delivery and behavioral control

Behavioral control and data acquisition was computer-controlled using the custom Voyeur software^36^. For odor stimulus delivery, we used an 8-odor olfactometer (Supplementary Fig. 1A). Odorants were diluted in mineral oil and stored in amber volatile organic analysis vials. Olfactometery manifolds, valves, and tubing contacting odorized air consisted entirely of PTFE to minimize cross-contamination of odorant. During odor presentation, nitrogen carrier gas is diverted through a single vial and enters the main air stream, resulting in an adjustable dilution in a range between 10× and 100×. Airflow rates for carrier and main flow rates were controlled using two mass flow controllers (Bronkhorst). During periods between stimuli, animals were presented with 1 L/min background air and olfactometer air was directed to exhaust using a four-way PTFE final valve (NResearch).

Odorant concentrations were controlled using a combination of gas- and liquid-phase dilution. Through manipulation of the ratio of odorized and clean air flow rates, we were able to achieve a dilution range of 10–1% odorized air (10×). In experiments where more that 10-fold dilution was required, we used liquid dilution to increase our range. Because liquid dilution ratios do not accurately predict headspace (gas-phase) concentrations, liquid dilutions were assayed using a photo ionization detector (Aurora Scientific) to determine relative concentrations of odorant in gas-phase. For concentration-series electrophysiology, all dilutions were made in air-phase by diluting odorized air with unodorized air.

Mixtures of carvone enantiomers were made in liquid phase. Enantiomers have identical vapor pressures and solvent interactions, thereby allowing accurate prediction of component ratios in gas phase from liquid mixture ratios.

Light masking stimulus was provided by two 473-nm, 105 um ID fiber coupled diode laser (Blue Sky Research, PN: FTEC2471) terminated in a ceramic ferrule. During behavioral sessions, the laser source ferrule was mated to a ferrule permanently implanted on the mouse. Implanted ferrules (MM-FER2007-304-4050, Precision Fiber Products, Milpitas, CA) were fabricated with 400 um ID, 0.39 NA fiber (FT400UMT, Thorlabs, Newton, NJ) and etched using hydrofluoric acid to provide diffuse light within the nasal cavity. Laser power was calibrated using a light power meter (Thorlabs) prior to behavioral sessions at the ferrule tip.

Water reward stimulus was delivered through two 21-gauge stainless steel lick tubes (Small Parts) and controlled using pinch valves (BioChem Fluidics).

Licks were detected by measuring change in resistance at the lick port when animals made contact with the lick tube using custom lick detectors (Janelia, HHMI).

### Behavioral procedure and training

Animals were water deprived for at least 5 days prior to start of behavioral training. Animals were housed on a 12:12 light–dark cycle were tested between 1500 and 2400 ZT (where ZT 0 corresponds to beginning of light period). To acclimatize animal to head-fixation and behavioral apparatus, animals were shaped by given water through a single lick tube until animals received their entire 1 ml water ration during a session. In subsequent sessions, a second lick tube was introduced. To encourage exploratory behavior in subsequent training, animals were rewarded for alternating licks between left and right lick tubes. Two-lick shaping sessions persisted until animals successfully received entire 1 ml water ration in a session.

Odor discrimination was trained on subsequent sessions with only slight modifications to the paradigm used in testing sessions (Supplementary Fig. 1C). After a variable inter-trial interval (12–15 s) and with the condition that 1 s had elapsed since the last licking activity was recorded. Odor stimulus was delivered for 500 ms and was initiated on the start of exhalation so that odor stimulus was stable prior to inhalation (Supplementary Fig. 1B). For each trial, odor concentration was drawn randomly. After stimulus onset, a “grace” period was enforced where licks were not scored to reduce impulsive licking prior to odor sampling. To eliminate stereotypic response bias, trials were chosen using a bias correction algorithm during training and testing^37^. For initial training, this grace period included time point until 500 ms after the first inhalation of odorant. After criterion performance was met, this grace period was shorted in the following session to 300, then to 150 ms for testing. Randomization was preformed using Mersenne Twister RNG (NumPy).

Testing sessions were conducted only after animals reached criterion performance (>80%) on the odor discrimination task. Mask trials was randomly interleaved in sessions for only 2 of the 5 concentrations presented (Fig. 2a). Trial ordering within a session was computer-controlled and the investigator was blind to the conditions of each trial. For masking data at multiple concentrations, masking trials was presented in every other session with both concentrations masked in the same sessions at rate of 17% of total trials. For the masking data for carvone mixtures, masking trials were interleaved throughout each session at a rate of 8.3% of total trials. The data were excluded from animals that did not complete training and testing due to loss of sniff signal, illness, or loss of implant. Two animals were excluded from mixture experiment due to faulty fiber implantation. The data from experiments represented in Fig. 3b and Supplementary Fig. 1D were collected from the same animals.

### Behavioral analysis

All behavioral analysis was conducted using custom scripts on the Anaconda Python distribution (NumPy 1.9.2, SciPy 0.15.1)^38^. Binomial proportion confidence intervals were calculated using the Clopper–Pearson “exact” method. Trials from sessions in which animals preformed a level of <80% correct responses were excluded from analysis. The data were fit with the Weibull psychometric function using maximum likelihood estimation method:

$${p_x} = \frac{{\gamma + \left( {1 - \lambda } \right)}}{{1 + {e^{ - \beta \left( {x - \alpha } \right)}}}}$$

For the masking data, *γ* (guess rate: the asymptotic performance at short latencies) was fixed using the average from masking at time points < 60 ms and *λ* (lapse rate: the asymptotic performance at long latencies) was fixed based on the data obtained in unmasked trials within these sessions. For reaction time analysis, all parameters were fit. Confidence intervals for fit parameters (thresholds) were estimated using the 2.5th and 97.5th percentile of distributions created by fitting each of 10,000 bootstrap simulations for each experimental condition. To bootstrap, trials were randomly drawn with replacement using Mersenne Twister RNG (Numpy).

The reaction time data were taken only from trials in which no mask stimuli were presented. These sessions were interleaved with mask sessions. The reaction time performance data and timing were taken using the first responses following odor stimulus onset irrespective of grace period. These data were fit using the same techniques as above, but with all parameters free. Trials with very long reaction times ( > = 300 ms) were truncated from this analysis, as performance was not monotonic after ~300 ms.

### Masking electrophysiology

Electrophysiology was conducted in awake animals during using acute recording techniques. Six-shank, 64-ch silicone probes (Buzsaki 64sp, NeuroNexus) were used to record neural activity. The neural data were acquired using “Whisper” acquisition system (Janelia, HHMI) at 20,833 Hz using SpikeGL software (Janelia, HHMI). Action potentials were detected and clustered using SpikeDetekt2 and KlustaKwik with manual clustering preformed using KlustaViewa^39^.

All basic analysis was done using the Anaconda Python distribution. Mask response latency was determined by comparing baseline (no mask) activity distribution to mask response. To construct baseline sample distribution, PSTHs for 7 sniffs prior to every mask trial were sampled. From these baseline samples, 100,000 samples of the same size as the number of mask trial were drawn with replacement to create a simulated PSTH from the same number of trials as the masked PSTH. Finally, the PSTH from masked trials was compared with the baseline PSTH distribution. Latency to mask response was defined as the first bin where firing rate was > 3 fold greater than the baseline PSTH and the bin was at the 0.0001th percentile of the bootstrapped baseline distribution.

### Linear SVM classifier

Population vectors were assembled from spike trains of recorded unit (*n* = 29) that responded to one of the odors presented. For each cell and for each trial, a 35-dimensional vector was created by binning action potential events into 10 ms bins from the 0–350 ms after the first inhalation onset of the odorant. Activity vectors from cells were concatenated and standardized to make a population vector for training and testing. Linear classifiers were created using the Scikit-learn v0.16.1 LinearSVC class^40^. To assess performance of the classifiers on unmasked trials, a leave-one-out cross validation strategy was used. To classify masked trials, classifiers were built using all available unmasked trials (*N* = 19), and the classifier was scored on its performance at classifying all masked trials.

### Concentration series electrophysiology

NeuroNexus A64 Poly5 2 × 32 probes were used to record acutely from awake animals. Units were detected using Spyking Circus software (v0.3.0)^41^. Significant odor responses were defined by comparing inhalation-aligned blank odor response distributions with responses to odor presentations (Supplementary Fig. 3A–D). To establish these distributions, blank and odor responses were bootstrapped across trials, summed across trials, and smoothed with a 3-sigma Gaussian kernel of width 30 ms. For each time point, the bootstrapped firing rates were fitted with a Gaussian and the Cohen’s D (discriminability) score was calculated for each time bin. Response latencies were defined as the first time-bin in with a D-score above 5, where overlap is < 0.01 (Supplementary Fig. 3E, G).

### Computational model

Our model was based on random and sparse connectivity between the MT cells and the PC cells, as well as within the cortex as detailed below. Our simulation included 300 MT cells and 1000 cells in the PC. Neurons in PC formed random sparse inhibitory recurrent connections to other cells in PC with 50% probability. Non-zero inhibitory recurrent connection weights within PC were $W_{ij}^{inPP} = 3$. MT cells also formed random excitatory connections with the PC cells with 13% probability (each PC cell received inputs from 40 MT cells). Non-zero projections from MT cells to a PC cell were $W_{ij}^{inPB} = 0.15$. In the case of order dependent model, we used $W_{ij}^{inPB} = 0.30$.

The state of each PC neuron was defined by the input that this neuron receives *u*_*i*_ that satisfied the equation

$$\tau {\rm{d}}{u_i}/{\rm{d}}t = - {u_i} + \mathop {\sum }\limits_j W_{ij}^{PP}{f_j} + \mathop {\sum }\limits_j W_{ij}^{PB}{m_j}$$

Here *τ* = 0.05 is the time constant measured in the fraction of sniffing cycle. The activation state for each PC neuron had a hysteretic dependence on its inputs *f*_*i*_ = *F*_±_(*u*_*i*_). The activation function *F*_±_ was single valued for values of input variable *u* satisfying *u* > *u*_+_  = 0.2 and *u* < *u*_−_ = −150. For these values of parameters, the activation function *F*_±_ was equal to 1 and 0, respectively. Within the bistable range, i.e., for *u*_−_ ≤ *u* ≤ *u*_+_, *F*_±_ was bistable and remained constant depending on prior history. Therefore, if a neuron was activated, the activation function within the bistable range remained equal to 1, whereas for an inactivated neuron, the activation function was 0. Activation occurred when inputs exceeded *u*_+_, and inactivation happened when inputs fell below *u*_−_.

Each simulation was carried out over the period of 700 time units using Runge–Kutta method with time step Δ*t* = 0.002. The simulation started at *t* = −0.2 and lasted until *t* = 1.2. *t* = 0 corresponds to the onset of inhalation, and *t* = 1 is the end of the early stage of the sniff cycle. The masking stimulus was presented between *t* = 0 and *t* = 1. This time interval was expected to reproduce the early part of the sniff cycle during which odorant identity is established.

To model MT cell responses to odorants, we generated random spatiotemporal patterns of MT cell activity. For the case of pure odorants (black and gray in Fig. 5d), we used two different random patterns for each of the two odorants. The response of each responsive MT cell was represented by a transient that lasted 0.5 time units (in fractions of the early part of the sniff cycle). The transient response consisted of an increase of the output of a mitral cell *m*_*j*_ from 0 to 1 and reset back to 0. For low/high concentration conditions, the earliest transients began at *t* = 0.4/0.25, respectively and the following MT cell transients were distributed at random times with the time step of 0.02 (every 0.02 a MT cell was recruited). This feature was intended to replicate the tendency of MT cells to respond later in the sniff cycle to lower odorant concentrations^42^. For the mixture case (Fig. 5d), we generated two sets of random recruitment orders for each of the pure binary compounds within the mixture and combined them into a single sequence by offsetting one of them by 2 positions, depending on which component concentration was bigger (60 + 40% vs. 40 + 60% composition). Following the earliest transient onset at *t* = 0.25, as in high concentration case, one MT cell was recruited every time interval of 0.02 with the recruitment order as described above. In each trial, MT cell transients had a finite probability to be emitted to mimic the experimentally observed transient event reliability^43^. The probability was *p* = 0.8/0.9 for low/high concentration conditions. To simulate the ChR2 stimulation, we added a pulse that began at the time indicated in Fig. 4 and lasted 0.1. The amplitude of the pulse was 0.18. The pulse was present in 75% of MT cells chosen randomly. We added normally distributed white noise with the standard deviation of 0.1 to the activity of MT cells. This was done to reproduce observed features of psychophysical performance. We tested our simulations for a range of parameters and verified that the qualitative conclusions are not sensitive to the exact set of parameters chosen. We ran 500 trials for each of the two odorants and each concentration. After each set of 10 trials, we reset the randomly selected weights and parameters in the model to mimic trials performed by different animals.

The perceived identity of the stimulus in each trial was inferred from the activity of PC cells at the end of the simulation (*t* = 1.2) from the template pattern of PC response that maximally overlapped the evoked response. To obtain the template, we ran the simulation once without noise for every condition.

### Code availability

The code used for data acquisition is available at https://github.com/olfa-lab. MATLAB code of the computational model is available in ModelDB at http://modeldb.yale.edu/231814

### Data availability

The behavioral data set is available at https://doi.org/10.6084/m9.figshare.5325310. Concentration-series electrophysiological (spike times) data set is available at https://doi.org/10.6084/m9.figshare.5325340. Raw binary electrophysiological data will be made available on request to corresponding author.

## Electronic supplementary material

Supplementary Information
