# Supplementary material for: A primacy code for odor identity
Source: Nat Commun. 2017 Nov 14;8:1477. doi: 10.1038/s41467-017-01432-4 (PMC5684307; doi:10.1038/s41467-017-01432-4)
Supplement: Supplementary file 1 — Supplementary Information [file 41467_2017_1432_MOESM1_ESM.pdf]

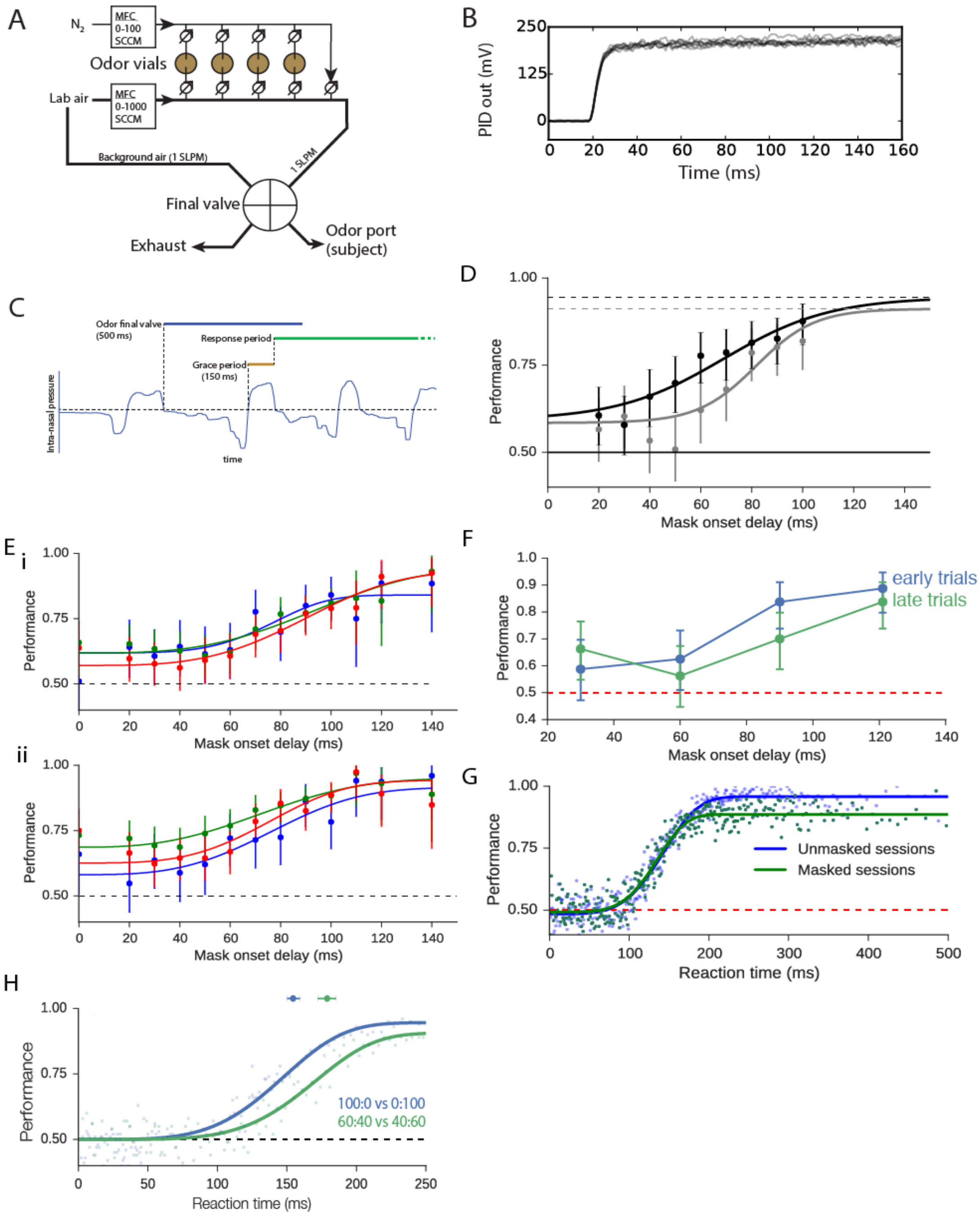

Supplementary Figure 1. Behavioral apparatus and experiments.

A. Olfactometer schematic. Illustration of 4-vial olfactometer. Liquid-diluted odorant is carried by nitrogen gas (N_2) flow into parallel carrier air stream when two vial valves are opened. Only one vial is exposed to nitrogen/air streams at a time to prevent cross-contamination. Sum of N_2 and air streams is 1 SLPM controlled by 2 mass flow controllers (MFCs), and adjusting ratio of these gasses controls dilution of odorant. Final valve (FV) positioned downstream of olfactometer is used to ensure fast and stable onset of odorant and continuous flow 1 LPM air for subjects.

B. Photoionization detector trace of odor time course demonstrates fast and repeatable odor presentations. FV is triggered on at t=0. D) Subjects to first inhalation following FV onset. Less than 0.5% of trials (*n* = 66,765) had inhalation before odor was available to the subject (30 ms).
C. Schematic of 2AFC behavioral trial. Behavioral trials are initiated with odor final valve opening at the start of the first exhalation following inter-trial-interval. Lick responses from are recorded but ignored until 150 ms after the initiation of first inhalation after odor valve trigger (grace period). Response period is relatively unrestricted (total trial time 2.5 seconds).
D. Performance vs mask onset delay for additional odor set (limonene vs pinene) at two concentrations: 0.1% (grey) and 1% dilution (black). Mask parameters were (100x1ms pulse, 200 Hz, 25 mW) for these experiments. Data were pooled for 4 subjects. Error bars express 95% confidence intervals.
E. Data and Weibull fits for individual subjects from Figure 3B for low (i) and high concentrations (ii) for experiments used for Figure 3.
F. Comparison of performance on early and late masking trials. The first 20 and last 20 masked trials for each animal at different delay time points were analyzed. Because of a low number of trials, data were pooled for all subjects (n=4) and data were binned for time points within 30 milliseconds. X axis value denotes the high value of the bin for each data point.
G. Comparison of reaction time to performance for sessions with and without masked trials.
H. Performance versus reaction time for pure carvone discrimination and carvone mixture discrimination.

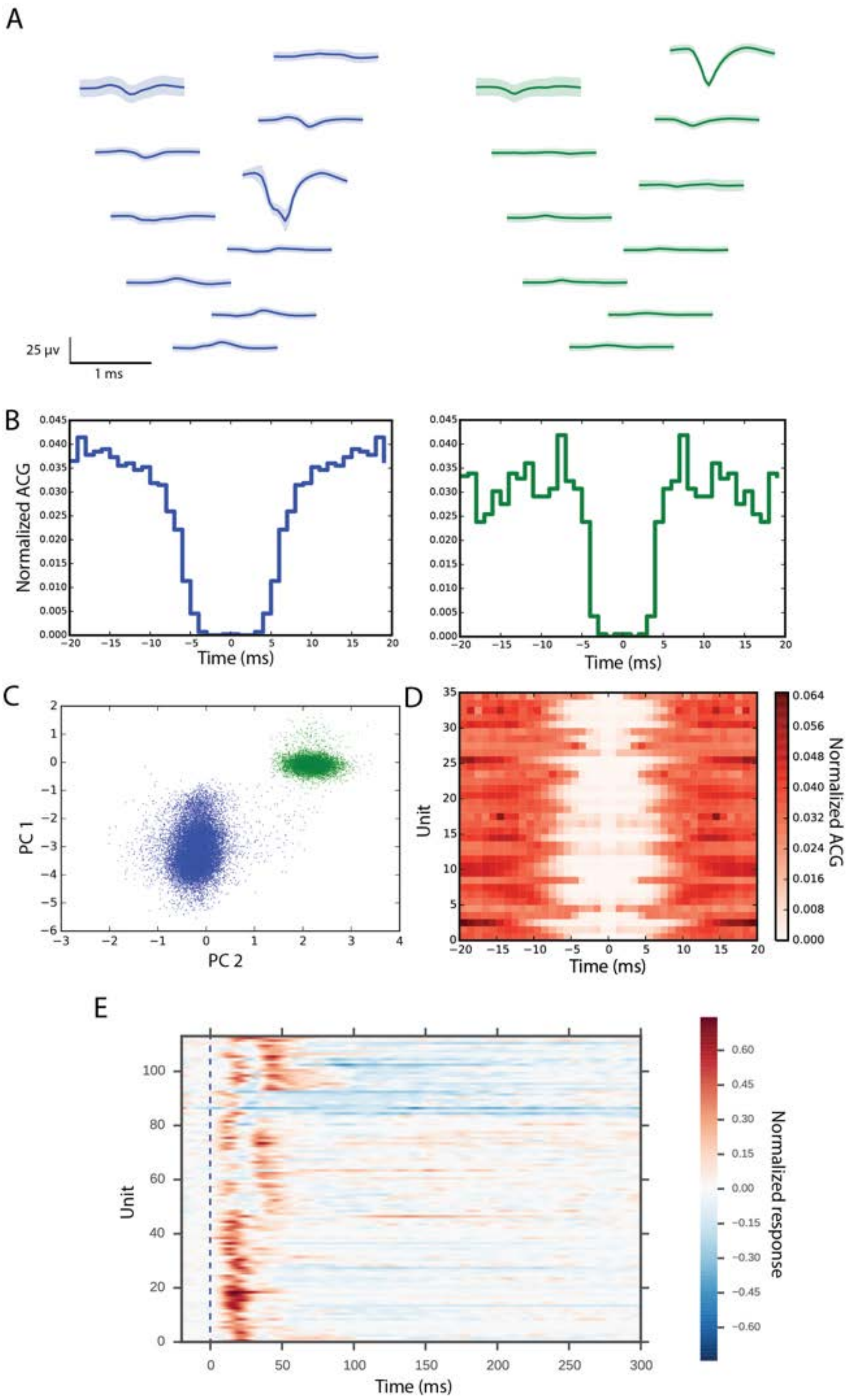

Supplementary Figure 2. **Masking electrophysiology supplementary information.**
A. Average waveforms of two single units (SU) recorded units recorded on the same electrode shank. Waveforms are displayed for all of the electrode sites on the shank in approximate relative position and different cells are denoted by color. Error estimation is standard deviation from the mean trace.

B. Autocorrelelograms (ACGs) for example units from A. ACGs are normalized based on the sum of the ACG from 0 to 20 ms.
C. Projection of waveforms on 2 principle components used for sorting. D) ACGs for each SU used for analysis displayed as heat map. ISI ratio ($ISI_{0\text{-}2ms}/ISI_{0\text{-}20ms}$) for all SU were < 0.1.
E. Peristimulus time histogram response to short-mask (2 x 2 ms pulses) for all units recorded. Laser stimulation initiated at t = 0 ms. PSTHs are baseline subtracted, normalized to their maximum response bin value, and smoothed by convolving 1 ms binning with a Gaussian (σ = 4 ms).

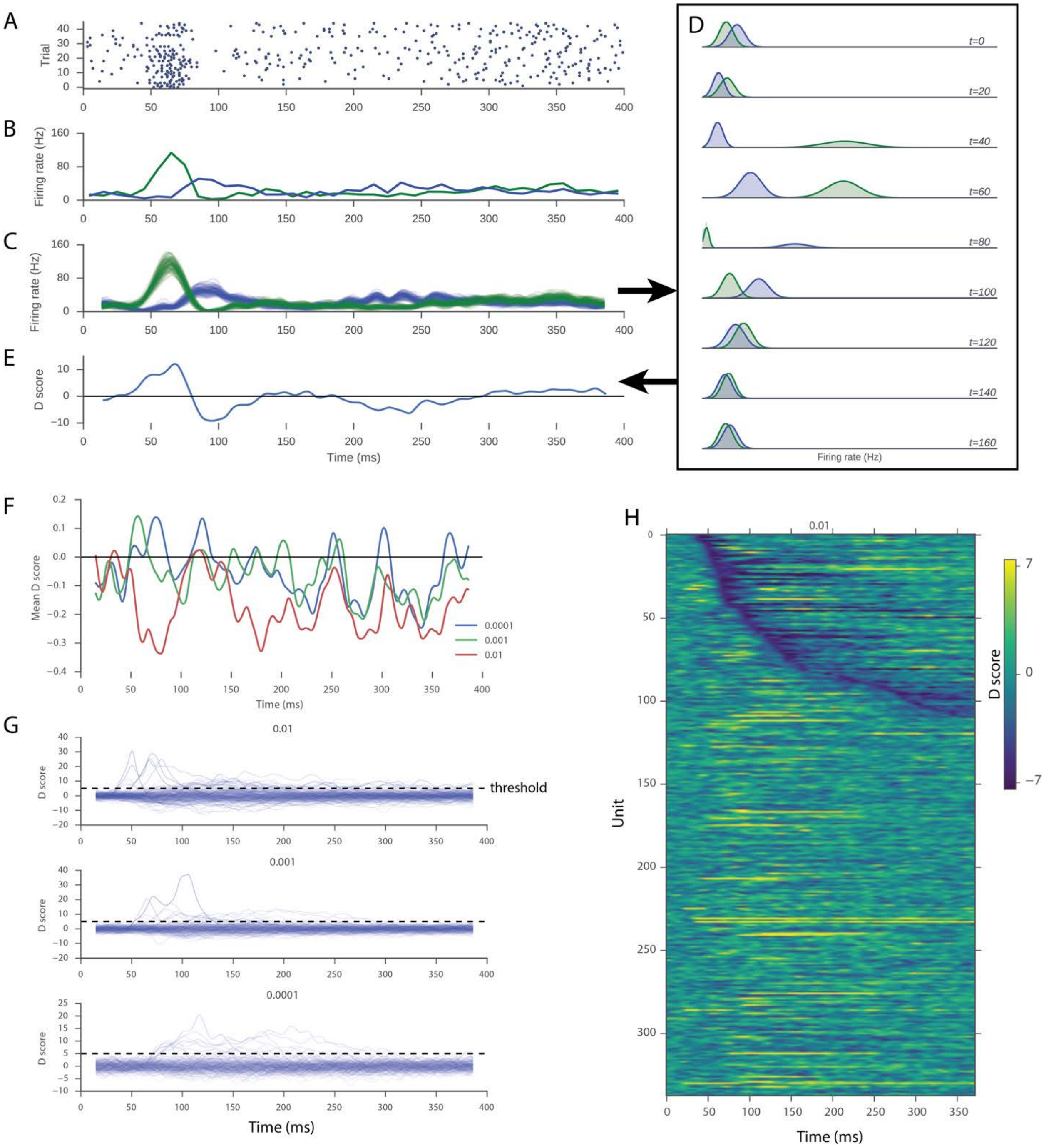

Supplementary Figure 3. Defining odor response latencies across concentration.

A. Raster of odor responses for example unit. Time 0 is defined as inhalation onset.

B. PSTH plot from raster. Odor response PSTH is shown in green and blank response in blue.

C. Bootstrapped PSTH plots. Each PSTH instance was created by sampling with replacement from trials, summing the responses across trials for each bin, and convolving a Gaussian kernel over this array (see methods).

D. Histograms of bootstrapped responses for odor and blank trials at different time points relative to inhalation onset. Fits of normal distributions used to calculate D-scores are overlaid.

E. D-score over time. Positive values specify excitation (odor response distribution is greater than blank distribution) and negative values specify inhibition relative to unit's blank response.
F. Mean D-score odor response across all units for each concentration tested. Note early inhibition at highest concentration.
G. Individual unit D-score odor responses for all units. Each axis represents responses at the concentration specified above the axis. The threshold used to determine response latencies is overlaid as a dashed line. The threshold is constant across concentrations.
H. D-scored responses of recorded population for one concentration of odorant. Units are sorted by latency to inhibitory response. Latency is determined by the first time point in which each units' odor response D-score crossed a threshold of -5.

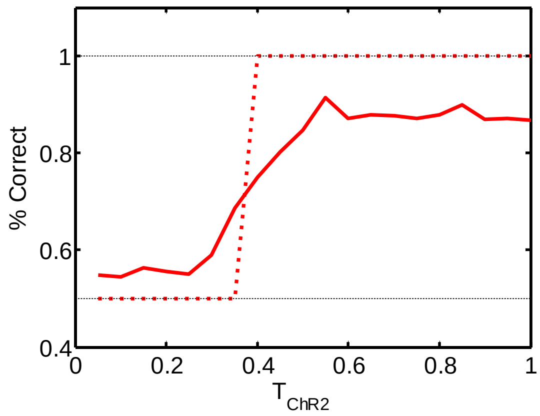

Supplementary Figure 4. In our computational model, an incomplete suppression of animals' behavior to 50% by the masking stimulus at short masking times is due to sources of variability and noise. Solid red line: same as in Figure 5D. Dotted figure, the same with no neural noise and for a single network weight configuration (emulating the conditions of a single animal). The latter modification was made to eliminate the variability due to varying performance in different animals. The behavior as affected by the mask jumps from pure 50% and 100% supporting the interpretation that, in the model, the deviations from pure 50% performance are due to internal sources of noise and individual variabilities.

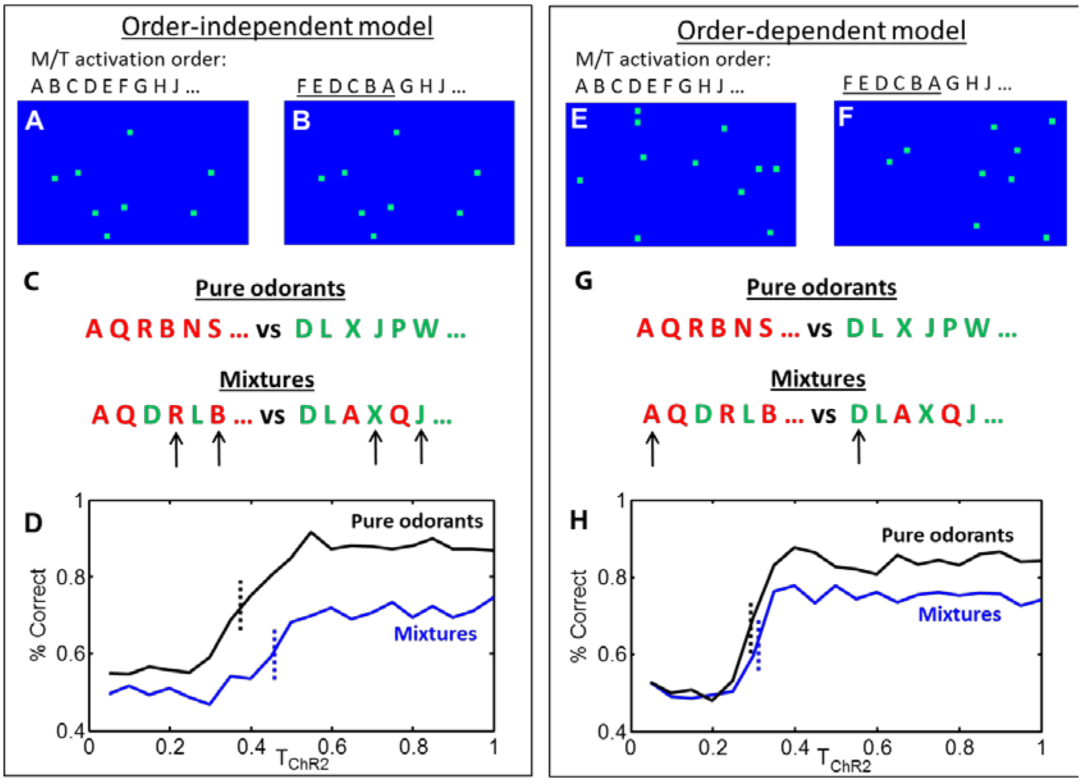

Supplementary Figure 5. Order dependent and order independent primacy models give different predictions for the dynamics of masking response.

A-B. Order-independent model. The patterns of PC activation are the same for different MT activation orders (letters on tops of panels), differing in the early stages of the sequence (differences underlined). These two glomerular activation sequences were chosen as examples to demonstrate the recruitment order independence of this instance of the model.

C. Glomerular activation sequences in the modelling of masking response. To test the performance of the model in response to mask, we presented two different random sequences for pure odorants (top). For the case of mixtures, the recruitment order was obtained by mixing two pure odor sequences with the temporal offset equal to two positions. The sequence corresponding to the lower concentration component (40%) was delayed w.r.t. the higher concentration sequence (60%). Arrows show differences between pools of activated glomeruli. They occur later in the sequence (4th and 6th positions) delaying the effect of mask in the case of similar mixtures.

D. Order independent model displays a small delay in the masking response for the case of mixtures (blue vs. black dotted lines).

E-F. Order dependent model displays differing patterns of PC activation when the order of MT cell activation is different. To implement the sensitivity of the model to the order of activation sequence, we increased the strength of PB->PC weights from 0.15 to 0.3. This makes PC start detecting coincidences earlier in the sniff cycle.

G. The activation sequences in this model were taken to be the same as in the order-independent model. Yet, the differences in the activation sequence emerge earlier in the sniff cycle (arrows) leading to earlier psychophysical performance.

H. The order-dependent model shows a much smaller difference in the timing of behavioral responses to mask. The comparison of order-independent and order-dependent models suggests that the slight

delay observed in the performance in similar mixtures of enantiomers (Fig. 3H) may be due to a form of order-independence in the decoding of the primacy sequence.
